# Effects of forest bathing on pre-hypertensive and hypertensive adults: a review of the literature

**DOI:** 10.1186/s12199-020-00856-7

**Published:** 2020-06-22

**Authors:** Katherine Ka-Yin Yau, Alice Yuen Loke

**Affiliations:** 1 School of Nursing, Tung Wah College, 31 Wylie Road, Homantin, Kowloon, Hong Kong; 2 school of Nursing, The Hong Kong Polytechnic University, Kowloon, Hong Kong

**Keywords:** Forest bathing, Forest therapy, Forest walking, Shinrin-yoku, Pre-hypertensive, Hypertension, Mood states, Stress level

## Abstract

The aim in this literature review was (1) to explore the physiologically and psychologically therapeutic benefits of forest bathing on adults suffering from pre-hypertension or hypertension, and (2) to identify the type, duration, and frequency of an effective forest bathing intervention in the management of pre-hypertension and hypertension, so as to provide directions for future interventions or research. The electronic databases PubMed, Cochrane Library, CINAHL, PsyINFO, and the China Academic Journals (CAJ) offered through the Full-text Database (CNKI) were searched for relevant studies published from the inception of the databases to April 2019. Of the 364 articles that were identified, 14 met the criteria for inclusion in this review. The synthesis of the findings in the included studies revealed that forest bathing interventions were effective at reducing blood pressure, lowering pulse rate, increasing the power of heart rate variability (HRV), improving cardiac-pulmonary parameters, and metabolic function, inducing a positive mood, reducing anxiety levels, and improving the quality of life of pre-hypertensive or hypertensive participants. Forest walking and forest therapy programs were the two most effective forest bathing interventions. Studies reported that practicing a single forest walking or forest therapy program can produce short-term physiological and psychological benefits. It is concluded that forest bathing, particularly forest walking and therapy, has physiologically and psychologically relaxing effects on middle-aged and elderly people with pre-hypertension and hypertension.

## Introduction

Hypertension is a major risk factor for cardiovascular diseases, including coronary and cerebral vascular diseases [1, 2]. It is estimated that by 2025, more than 1.5 billion people worldwide will suffer from hypertension, and the estimated annual number of deaths related to high blood pressure will increase to 10.4 million [2]. Hypertension has been defined as consistently having systolic blood pressure (SBP) of 130-139 mmHg or higher and/or diastolic blood pressure (DBP) of 80-89 mmHg or higher, while pre-hypertension is defined as consistently having a systolic blood pressure of 120-139 mmHg or diastolic blood pressure of 80-90 mmHg [3, 4]. Providing a definition of pre-hypertension helps to alert adults living with pre-hypertension to be aware of their increased risk of developing hypertension and cardiovascular diseases [5].

Approaches such as the use of antihypertensive drugs and lifestyle modifications, such as lowering one’s intake of dietary sodium, increasing one’s levels of physical activity, and losing weight, are recommended to control high blood pressure and prevent cardiovascular complications [2, 6]. Hypertensive medications are costly. The US government is reported to have spent billions of dollars on antihypertensive drugs in 2012-2013 [7]. It has been reported that many patients do not take their medications on a regular basis or even stop taking their medications because of the side effects or high cost involved [1, 8]. Lifestyle modifications are considered a more cost-effective way of addressing hypertension, but such modifications require persistent effort and personal commitment [2, 9–12]. Controlling blood pressure so that it does not reach elevated levels may reduce the cardiovascular morbidity and mortality of pre-hypertensive or hypertensive patients [13]. However, most pre-hypertensive and hypertensive individuals are not aware of the risks of hypertension and are not making lifestyle modifications to control their blood pressure [7, 14, 15].

Forest bathing, referred to as “Shinrin-yoku” in Japanese, involves spending time in a natural environment or specifically in a forest environment to improve one’s health and well-being [16–18]. Forest bathing was first proposed in the belief that spending time in a natural or forested area will have healing effects [16]. Forest bathing, through exposure to natural stimuli such as plants, woods, and flowing water in a forest environment, is regarded as a health promotion strategy to achieve relaxation, resulting in a decrease in heart rate and blood pressure, a release of stress, and a boost to the immune system, all of which facilitate recovery from illness [19–21]. During forest bathing, individuals are guided to slow down their pace and are soothed through connecting with a forest environment, using their five senses to listen to birds chirping and streams flowing, looking at trees and seeing sunlight penetrate through tree branches, breathing in natural aromas, tasting the freshness of the air, touching leaves and trees, and lying on the ground [16].

In recent years, researchers from Japan, Korea, China, and Europe have become interested in conducting studies to assess the health benefits of forest bathing [18, 22–24]. Studies conducted in the USA [20, 25] have found that exposure to a forest environment could increase the activities of the parasympathetic nervous system, induce mental relaxation, and decrease the production of stress hormones. A previous review of 28 studies reported that forest bathing contributed to the improvement of depressive symptoms in adults [18]. Another review also reported on the potential therapeutic effects of exposure to a natural environment, which could induce protective effects against cardiovascular diseases and even cancer [20].

Studies conducted in Japan and Taiwan support the view that forest bathing has beneficial effects, by showing that it helps to increase the natural activities of killer cells, which enhances the function of the immune system and prevents the growth of tumors [26, 27]. It is said that exposure to nature or to forest stimuli can increase people’s awareness and increase activities in parasympathetic nervous system causing people feeling of relaxation as well as boost weakened immune functions to prevent illnesses or promote recovery [21, 28].

Stress in everyday life causes a consistent elevation of blood pressure, which contributes to hypertension [29]. In response to stressful situations, our body responds by increasing the release of stress hormones into the bloodstream, causing an increase in heart rate and blood pressure [30]. Reducing stress is one way of controlling hypertension, and exposure to a forest environment is considered helpful at reducing stress, promoting relaxation, and improving moods, resulting in a decrease in heart rate and blood pressure [19, 20, 31].

Studies have shown that participants who were exposed to a forest environment experienced a reduction in blood pressure, pulse rate, and heart rate compared to those exposed to an urban environment [23, 32–36]. A study conducted in Japan found forest bathing activities were effective at lowering blood pressure and improving negative emotions in working-age adults [37]. Five systematic reviews evaluated the effects of forest bathing on health and well-being [17, 18, 20, 38, 39]. One review of 28 studies assessed the effects of forest bathing and concluded that forest bathing was effective at reducing levels of depression in adults [18]. Another review of 20 studies compared the effects of a forest environment on hypertensive adults of different age groups. It was found that forest bathing was effective at lowering systolic blood pressure in hypertensive middle-aged or older people [17].

Three other reviews—one of six randomized controlled trials and another two of 68 studies and 28 studies—examined the multiple physiological and psychological benefits of spending time in a forest [20, 38, 39]. These three reviews reported that forest therapy had beneficial effects on blood pressure, heart rate, pulse rate, and immune function, led to a reduction in inflammation, a decrease in the secretion of stress hormones, an improvement in cardiopulmonary functions and endocrine function, and to a reduction in depression and improvement in mood [20, 38, 39]. However, these reviews concluded that evidence of the therapeutic effects of forest bathing was insufficient to establish clinical guidelines on its use as a health promotion or disease prevention strategy for preventive medicine.

While these reviews provided some evidence that forest bathing has positive effects on the physiological and psychological responses of adults, none focused on examining the types, approaches, duration, and frequency of the forest bathing activities that provided physiological and psychological benefits to pre-hypertensive or hypertensive adults. A review of the literature focusing specifically on pre-hypertensive or hypertensive adults is essential to provide the evidence that is needed if such a therapy is to be promoted as a preventive medicine strategy for these adults.

The aim in this literature review is (1) to explore the physiologically and psychologically therapeutic benefits of forest bathing on adults suffering from pre-hypertension or hypertension, and (2) to identify the type, duration, and frequency of an effective forest bathing intervention in the management of pre-hypertension and hypertension, to provide directions for future interventions or research.

## Method

### Search strategies

A search for literature was undertaken using the following electronic databases: PubMed, Cochrane Library, CINAHL, PsyINFO, and the China Academic Journals (CAJ) offered through the Full-text Database (CNKI). Studies published from the inception of the database to April 2019 were searched. Only those published in English or Chinese were retrieved. These five databases contain published studies covering the fields of biomedicine, healthcare, psychology, nursing, life sciences, behavioral sciences, medicine and health science, and rehabilitation sciences that could provide evidence on the topic of interest.

The search terms used to identify literature included “pre-hypertension” or “hypertension” AND “forest bathing” or “forest therapy” or “forest walking” or “forest environment” or “shinrin-yoku” AND “intervention” or “randomized control trials” AND “blood pressure” or “pulse rate” or “heart rate” or “stress level” or “mood status”. The reference lists of the retrieved studies were also searched for possible additional relevant studies. Full-text studies were then retrieved and evaluated for eligibility. This review followed the PRISMA (Preferred Reporting Items for Systematic Reviews and Meta-Analyses) guidelines.

### Selection criteria

The studies that were retrieved were screened based on the following inclusion and exclusion criteria. Included were studies that (1) focused on adults 18 years or older who were suffering from pre-hypertension or hypertension, (2) consisted of intervention studies or randomized control trials (RCT), with or without a comparison group, (3) involved an intervention such as forest bathing, forest therapy, forest walking, or a forest environment, and (4) evaluated blood pressure, pulse rate, heart rate, stress level, and mood status as outcomes. Excluded were studies that (1) included participants who were pregnant, had cancer, stroke, or mental illness, and (2) cross-sectional studies.

### Study selection process

A total of 364 studies were identified from the literature search. Nine were removed due to duplication. The remaining 355 publications were screened by title and abstract, and 219 studies were excluded. Publications were excluded if they focused on knowledge of hypertension (*n* = 59); examined the effects of an anti-hypertensive drug (*n* = 17); the participants were pregnant women (*n* = 10); focused on a pharmacological intervention or on a non-pharmacological intervention other than forest bathing for hypertension (*n* = 36); focused on adults with cardiovascular disease, diabetes, kidney disease, or cancer (*n* = 84); consisted of a review on the benefits of exposure to a forest environment (*n* = 4); and if no full-text version of the study was available (*n* = 9).

After screening titles and abstracts, 136 full-text studies remained for further evaluation based on the inclusion and exclusion criteria, and 125 studies were further excluded for the following reasons: the focus was on patients with chronic heart failure, chronic obstructive pulmonary disease, cancer, or chronic pain syndrome (*n* = 13); the participants were healthy university students or healthy middle-aged adults (*n* = 15); the study was a cross-sectional survey (*n* = 1); or the study consisted of a description of the beneficial effects of forest bathing published in Chinese (*n* = 96).

**Fig. 1:**
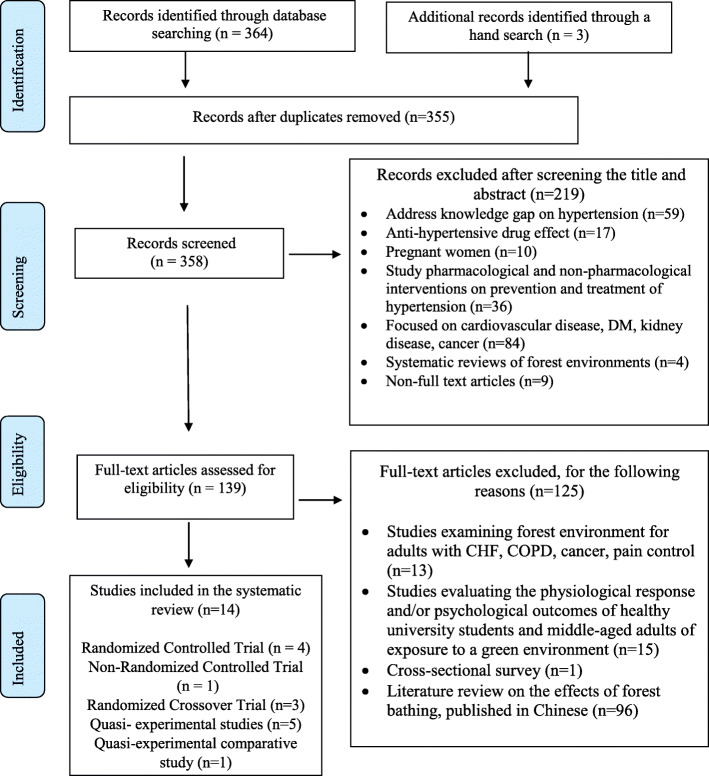
Flow chart of the literature search process (PRISMA 2009)

Finally, 11 articles were retrieved. Three additional studies were identified through a manual search of the reference list of the included studies. In the end, a total of 14 articles were included in this review. Two articles were published in Chinese and 12 articles were published in English. A flow chart of the literature search and selection process is given in Fig. 1.

### Assessing the quality of the literature for risk of bias

The 14 studies were independently appraised by two reviewers for risk of bias. The quality of the studies was assessed using the quality assessment tool for studies with diverse designs (QATSDD) [40]. This tool consists of 16 items, with scores ranging from 0 to 3 for each item. The criteria covered the requirements for the inclusion of an explicit theoretical framework; a determination of the size of the sample, the appropriateness of the study design, the data collection strategies, the statistical analyses, the reporting of the outcome measures, the involvement of the users; and a critical appraisal of the findings. Since two of the items in the QATSDD apply to qualitative studies, only 14 items were used to assess the quality of the included studies, with possible scores ranging from 0 to 42.

## Results

### Data extraction

A summary of the 14 included articles was tabulated according to the study characteristics (authors, year of publication, country where the study was conducted), study design, sample size, participants’ characteristics (age, sex), and the criteria for the inclusion of participants; the type of intervention (forest exposure and comparison intervention), the duration of the intervention, and the instrument(s) used to measure the state of mood of the participants (see Table 1). The physiological and psychological outcome measures of the interventions reported in the included studies are summarized in Tables 2 and 3 respectively.

**Table 1:** Characteristics of the included studies

Author, year, country	Study design	Sample size	Criteria for the inclusion of participants	Criteria for the exclusion of participants	Forest bathing intervention	Control/comparator	Duration and frequency of the intervention	QATSDD score (0-42)
Feng et al., 2017 [41]; China	Randomized controlled trial	*n* = 290, 100% male; *I* = 190 (HT group); *C* = 100 (non-HT group)	Mean age: 50 ± 10; non-smokers and non-drinkers; HT with or without anti-HTN drugs	DM; CAD; CVA	Forest walking	No intervention	20 sessions; 1 session/day; walk 60-90 min/per session or walk 2 km/per session	23
Horiuchi et al., 2015 [32]; Japan	Quasi-experimental study	*n* = 54; males = 19 (35%); females = 35 (65%)	Mean age: 63.2 ± 9.4; taking anti-HTN drugs; non-smokers	Not specified	Forest walking (stretching; self-paced, comfortable walking) and forest viewing in a supine position	NA	1 session; 90 min/per session	26
Lanki et al., 2017 [22]; Finland	Quasi-experimental and comparative study	*n* = 36; 100% female	Aged 30-60; not taking anti-HTN medications	Smoking; cardiac pacemaker; hearing aid; MI; CAD; CHF; Stroke, COPD	Sedentary viewing, and walking in an urban forest and urban park	Sedentary viewing and walking in the city center	1 session; starting in the afternoon; including 15 min of sedentary viewing, 30 min of paced and unhurried walking	25
Lee and Lee, 2014 [33]; Korea	Randomized controlled trial	*n* = 62; 100% female; I, *n* = 43; C, *n* = 19	Aged 60-80; BP < 160/110 mmHg	Chronic liver and renal disease; CAD; CVA; cancer; disability or pain when walking; BP > 160/110 mmHg	Forest walking conducted separately 1 week apart with city walking	City walking conducted separately 1 week apart with forest walking	1 session; 1 h of paced walking in the morning	31
Li et al., 2016 [34]; Japan	Randomized crossover trial	*n* = 19; 100% male	Aged 40-74; not taking anti-HT drugs; high normal hypertension; living in the city	Not specified	Forest walking	Urban walking	Two sessions in a day: (AM+PM); 1 h 20 min per session	23
Mao et al., 2012 [23]; China	Randomized controlled trial	*n* = 24; *I* = 12; *C* = 12; does not mention gender	Aged 60-75; BP < 180/110 mmHg with or without taking anti-HTN drugs; class I-II cardiac function; ADL independent	Getting the flu; acute disease 2 weeks before; cancer, chronic liver, kidney, brain, heart or lung disease; acute MI in the previous 3 months; CVA within 6 months; Hx of severe trauma or major surgery	Forest walking	City walking	7 sessions in 7 consecutive days, 1.5 h for each session, walk in the morning or afternoon	28
Ochiai et al., 2015a [24]; Japan	Quasi-experimental study	*n* = 9; 100% male	Aged 40-72; SBP 130-139 mmHg or DBP 85-89 mmHg	Taking drugs for DM, HT, hyper-lipidemia	Forest therapy: strolling; sitting; lying down; deep breathing in a forest; riding in the forest train; strolling in an indoor pavilion	NA	One-day therapy program, 4 h 35 min	28
Ochiai et al., 2015b [42]; Japan	Quasi-experimental study	*n* = 17; 100% female; HT (*n* = 6) healthy adults (11)	Mean age: 62.2+/−9.4; HT with or without anti-HTN drugs; no other diseases or psychological disorders	Difficulty walking in hot weather	Forest therapy: strolling, deep breathing, lying down lecture and chatting in forest; abdominal breathing with lie down position	NA	One-day therapy program, 4 h 41 min	28
Song et al., 2017a [35]; Japan	Randomized crossover trial	*n* = 20; 100% male	Aged 40-75; BP > 120/80 mmHg	Taking medication for diabetes, hyper- lipidemia, HT	Landscapes of forest viewed while sitting in chair in the afternoon	Urban area viewing while sitting in a chair in the afternoon	One session, 10 min each, conducted in 2 consecutive days	29
Song et al., 2017b [43] Japan	Quasi-experimental study	*n* = 26; males = 14; females = 12	Aged 19-56; office workers from an IT company; BP normal or SBP > 120 mmHg	Not specified	Forest therapy: preparation stretches; blind walking; deep breathing; strolling; viewing scenery and lecture; sending stress to waterfall, sitting and lying down; backwards walking; meditation; lying in a hammock	NA	One-day forest program, 6 h 12 min; date collected, 3 days before, on the day of the forest therapy, 3 days after, and 5 days after	24
Song et al., 2015 [36]; Japan	Randomized crossover trial	*n* = 20; 100% male	Mean age: 58 ± 10.6; BMI: 23.4 ± 3.3 kg.m2; SBP 130–179 mmHg; DBP 85–109 mmHg	Taking drugs for DM, hyperlipidemia, HT	Walking in a forest	Walking in urban area	One session, 17 min in 2 consecutive days	25
Sung et al., 2012 [44]; Korea	Non-randomized controlled trial	*n* = 56; I, *n* = 28; C, *n* = 28; females, *n* = 34; males, *n* = 22	I, mean age 66+/−7; C, mean age 63+/−11; SBP 130-159 mmHg or DBP 85-99 mmHg; on anti-HTN drugs	SBP > 159 mmHg or DBP > 100 mmHg; uncontrolled hypertension and need urgent change of drug regimen; comorbidity	Cognitive behavior-based forest therapy: HTN management, motivation to make therapeutic changes in lifestyle; practicing mindfulness relaxation techniques in the forest using the five senses	Printed educational materials for HTN management; self-monitoring of BP	3 days forest program with 8 weeks follow-up monitoring	27
Yu et al., 2017 [45]; Taiwan	Quasi-experimental study	*n* = 128; females, *n* = 85 (66.4%); males, *n* = 43 (33.6%)	Aged 45-86; chronic disease: DM, HT, heart disease, other disease	Not specified	Forest walking	NA	One session, 2 h starting in the morning	24
Zhou et al., 2017 [46]; China	Randomized controlled trial	*n* = 190 100% male; I, *n* = 95; C, *n* = 95	Average age: 50 years; diagnosed with HT	DM; CAD; CVA	Forest walking	Walking around the highway	20 sessions, each walk for 2 km; starting at 9:00 am	23

*HT* hypertension; *AM* in the morning; *PM* in the afternoon; *SDM* semantic differential method; *MI* myocardial infarction; *CVA* cerebrovascular accident; *POMS* the profile of mood states; *DM* diabetic mellitus; *COPD* chronic obstructive pulmonary disease; *CAD* coronary artery disease; *CHF* congestive heart failure; *HRV* heart rate variability; *I* intervention; *C* control; *MOS SF-36*:the medical outcomes study questionnaire short-form 36 health survey; *mHF* mean high frequency; *HRV* heart rate variability; *QATSDD* quality assessment tool for studies with diverse designs (Sirriyeh et al.)

**Table 2:** Summary of the physiological outcome measures of the included studies

Reference/participants	Change in SBP (mmHg)	Change in DBP (mmHg)	Change in heart rate (bpm)/HRV (Inms2)	Change in pulse rate (bpm)	Change in other outcome measures
Types of forest intervention: forest walking					
Feng et al., 2017 [41]; hyper-tensive middle-aged men	After forest walking, SBP was significantly lower by 13.2% in the drug group and 8% in the non-drug group.	After forest walking: DBP was significantly lower by 15.3% in the drug group and by 10.7% in the non-drug group.	NA	NA	After forest walking, total cholesterol, HDL, LDL, TG, IMT, and BaPWV improved in the drug group and non-drug group. FMD and NMD were improved remarkably in the drug group and non-drug group.
Lee and Lee, 2014 [33]; pre-hypertensive elderly women	Compared with city walking, SBP was lower by 10.26+/−13.11 mmHg (8.4%) after forest walks; compared with forest walking, SBP increased by 2.0+/−17.51 mmHg (2.6%) after city walks.	Compared with city walking, DBP was significantly lower by 9.93+/−11/15 mmHg (8.3%) after forest walking; compared with forest walking, DBP was unchanged after city walking.	NA	NA	Compared with city walking, CAVI was significantly lower by 0.42+/−0.72; and FEV1 and FEV6 were increased by 0.19+/−0.26 and 0.22+/−0.36, respectively compared with forest walking, CAVI, FEV1, and FEV6 were unchanged after city walking.
Li et al., 2016 [34] hypertensive middle-aged men	No significant difference in systolic blood pressure between forest and urban walking	No significant difference in diastolic blood pressure between forest and urban walking	NA	Forest walking significantly reduced the subjects’ pulse rate by 6.9%.	After forest walking, the serum level of adiponectin was significantly greater than that of urban walking. Forest and urban walks reduced the level of urinary adrenaline, noradrenaline, and dopamine but had no effect on total cholesterol, LDL, HDL, RLP, EIA, blood glucose, serum insulin, and DHEA-S, hs-CRP.
Mao et al., 2012 [23] hypertensive elderly people	After a 7-day forest walking trip, SBP decreased significantly by 8 mmHg (5.4%) compared with that of the city group; after a 7-day city walking trip, SBP showed little change compared with that of the forest walking group.	After a 7-day forest walking trip, DBP decreased significantly by 6 mmHg (7%) compared with that of the city group; after a 7-day city walking trip, DBP showed little change compared with that of the forest walking group.	Heart rate did not change in either of the two groups before and after the experimental intervention.	Pulse rate did not change in either of the two groups before and after the experimental intervention.	At the end of the 7-day trip, ET-1, Hcy, AGT, AT1, and AT2 levels were significantly lower in the forest group. There were no significant alterations in these factors in the city group. The serum IL-6 level in the forest group was lower in comparison with its baseline level.
Song et al., 2015 [36]; hypertensive middle-aged men	NA	NA	Compared with urban walking, mHF was 10% higher in forest walking; compared with urban walking, mHR was 1.9% lower in forest walking.	NA	NA
Yu et al., 2017 [45]; pre-hypertensive middle-aged and elderly people	Pretest: 129.9 ± 17.5 mmHg; post-test: 124.8 ± 16.5 mmHg; significantly lower by 3.9% after forest walking	Pretest: 85.3 ± 9.1 mmHg; post-test: 84.0 ± 8.1 mmHg; significantly lower by 1.5% after forest walking	No significant change in HF and LF/HF	Pretest: 73.9 ± 9.4 bpm; post-test: 71.4 ± 8.4 bpm; significantly lower by 3% after forest walking	NA
Zhou et al., 2017 [46] hypertensive middle-aged men	After forest walking, SBP decreased by 24.6%; after walking around a highway, SBP decreased by 17%.	After forest walking, DBP decreased by 29.5%; after walking around a highway, DBP decreased by 2%.	After forest walking, HR decreased by 28%; after walking around a highway, HR decreased by 20%.	NA	After forest walking, triacylglycerol, total cholesterol, and cardiac functions improved significantly compared with walking around a highway.
Types of forest interventions: sitting and viewing of landscapes in the forest					
Song et al., 2017a [35]; hypertensive middle-aged men	NA	NA	Compared with the urban area, HRV was significantly higher by 30% in forest viewing; no significant difference between the two environments in LF/HF; compared with the urban area, HR was significantly lower by 3.5% in forest viewing	NA	NA
Types of forest interventions: forest walking and viewing of landscapes in the forest					
Lanki et al., 2017 [22]; pre-hypertensive middle-aged women	Walking in an urban forest was associated with a 1.9% increase in SBP; sitting and viewing the landscape in an urban forest was associated with no change in SBP; viewing and walking in an urban forest was associated with no change in SBP compared with viewing and walking in an urban park and in the city center	Walking in an urban forest was associated with a 2.5% increase in DBP; sitting and viewing the landscape led to lower DBP compared with sitting and viewing the landscape in an urban park and in the city center; viewing and walking in urban forest was associated with no change in DBP compared with viewing and walking in an urban park and in the city center	Walking in a forest was associated with a 5.4% lower heart rate and a higher HF by over 100%; viewing of landscapes in a forest area was associated with a 6.5% lower heart rate and a 25% higher HF after the intervention.	NA	NA
Horiuchi et al., 2015 [32]; hypertensive middle-aged and elderly people	After forest walking, SBP decreased significantly by 11% and 5% in the responder group (> 5% MAP decrease) and non-responder group (< 5% MAP decrease), respectively	After forest walking, DBP decreased significantly by 5% in the responder group (> 5% MAP decrease) but was unchanged in the non-responder group (< 5% MAP decrease)	NA	NA	No improvement in salivary amylase (sAmy) was observed before and after forest walking in both the responder group and the non-responder group
Types of forest interventions: forest therapy program					
Ochiai et al.,2015b [42]; hypertensive middle-aged women (*n* = 6)	NA	NA	NA	Pretest: 73.1 ± 2.5 bpm; post-test: 69.1 ± 2.7 bpm; significantly lower after forest therapy by 4.67 bpm (5.4%)	Salivary cortisol concentration: pretest: 0.168 ± 0.020 μg/dL; post-test: 0.124 ± 0.009 μg/dL; significantly lower after forest therapy by 2.63 μg/dL
Ochiai et al., 2015a [24]; pre-hypertensive middle-aged men	Pretest: 140.1 mmHg, post-test: 123.9 mmHg; significantly lower by 16.1 mmHg (11.5%)	Pretest: 84.4 mmHg; post-test: 76.6 mmHg ; significantly lower by 7.8 mmHg (9.2%)	NA	NA	Salivary cortisol concentration, pretest: 7.4 μg/dL; post-test:4.9 μg/dL significantly lower after forest therapy by 2.5 μg/dL; urinary creatinine correction, pretest: 13.1 μg/g creatinine; post-test: 11.0 μg/g creatinine; significantly lower after forest therapy by 2.1 μg/g creatinine
Song et al., 2017b [43]; pre-hypertensive middle-aged adults; HT, *n* = 9, non-HT, *n* = 17	5 days after: decreased significantly by 3.5% (pre: 114.8 ± 2.7 mmHg; post: 110.7 ± 2.6 mmHg); higher than 120 mmHg group (*n* = 9): 5 days after: decreased significantly by 5.1% (pre: 128.4 ± 4.9 mmHg; post: 121.8 ± 4.6 mmHg)	5 days after: Decreased significantly by 2.8% (pre: 75.0 ± 2.3 mmHg; post: 72.9 ± 2.1 mmHg); higher than 120 mmHg group (*n* = 9),5 days after: decreased significantly by 5.3% (pre: 86.6 ± 3.4 mmHg; post: 82.0 ± 3.4 mmHg)	NA	Pulse rate: no significant change	NA
Sung et al., 2012 [44]; hypertensive elderly people	8 weeks after: I, −12.0 ± 9.2 mmHg; C, 11.5 ± 19.9 mmHg; decreased by 9 % from the initial measurement in the forest group compared with the control group	8 weeks after: I, no change; C, 1.3 ± 13.3; DBP did not show a significant change from the baseline, and self-measured SBP and DBP at week 4 and week 8 did not differ from the baseline measurements.	NA	NA	Salivary cortisol level: significantly reduced in the forest group by 0.03 μg/dL; in the control group, the salivary cortisol level increased slightly at the follow-up.

*FMD* flow mediated dilation; *NMD* nitro-glycerine mediated dilation; *IMT* carotid intima media thickness; *BaPWV* brachial-ankle pulse wave velocity; *TG* triglycerides; *LDL* low density lipoprotein; *HDL* high density lipoprotein; *RLP* remnant-like particles; *DHEA-S* the serum level of dehydroepiandrosterone sulfate; *hs-CRP* the serum level of high-sensitivity C-reactive protein; *GH* general health, *PD* physical dimension; *MD* mental dimension; *SD* social dimension; *HTN* hypertension-related dimension; *CAVI* cardio-ankle vascular index; *FEV1* forced expiratory volume in 1 s; *FEV6* forced expiratory volume in 6 s; *ET-1* endothelin-1, *Hcy* homocysteine, *RAS* renin-angiotensin system; *AGT* angiotensinogen; *Ang II* angiotensin II; *AT1* angiotensin II type 1 receptor, *AT2* angiotensin II type 2 receptor; *IL-6* the production of interleukin-6 and *TNF-α* tumor necrosis factor-alpha; *HR* heart rate; *LF/HF* low frequency/high frequency; *mHF* mean high frequency

**Table 3:** Summary of outcomes measures on psychological response of the included studies

Reference/participants	Change in mood states	Change in quality of life (QoL)/anxiety level measures
Types of forest interventions: forest walking		
Feng et al., 2017 [41]; hypertensive middle-aged men	NA	NA
Lee and Lee, 2014 [33]; pre-hypertensive elderly women	NA	NA
Li et al., 2016 [34]; hypertensive middle-aged men	POMS scores indicated a significant increase in positive feelings (vigor) and a significant decrease in negative feelings (tension, anxiety, depression, confusion, fatigue) after forest walking. POMS scores indicated a significant decrease in positive feelings (vigor) and a significant increase in negative feelings of fatigue after urban walking.	NA
Mao et al., 2012 [23]; hypertensive elderly people	POMS scores indicated a significant increase in positive feelings (vigor) and significant decrease in negative feelings (anxiety, depression, confusion, fatigue, anger) after forest walking compared with the baseline. POMS scores indicated a significant decrease in positive feelings (vigor) and no significant increase in negative subscales after urban walking.	NA
Song et al., 2015 [36];hypertensive middle-aged men	POMS scores were significantly higher for positive feelings (vigor) and significantly lower for negative feelings (anxiety, depression, confusion, fatigue, anger) after forest walking than after urban walking. SDM score: Participants felt more “comfortable,” “relaxed,” and “natural” when they walked in a forest area than in an urban area.	NA
Yu et al., 2017 [45]; pre-hypertensive middle-aged adults and elderly people	POMS score: There was a significant increase in positive feelings (vigor) and a significant decrease in negative feelings (tension anxiety, fatigue, anger, depression, confusion) after forest therapy.	STAI was taken before and after the intervention STAI: State anxiety subscale A decrease in score of 2% represented a significant improvement in anxiety levels
Zhou et al., 2017 [46]; hypertensive middle-aged men	NA	NA
Types of forest interventions: sitting and viewing of landscapes in a forest		
Song el at., 2017a [35]; hypertensive middle-aged men	SDM: Viewing in a forest area felt more comfortable, relaxed, and natural than in an urban area	NA
Types of forest interventions: forest walking and viewing of landscape in a forest		
Lanki et al., 2017 [22]; pre-hypertensive middle-aged women	NA	NA
Horiuchi et al., 2015 [32]; hypertensive middle-aged and elderly people	POMS score: There was a significant increase in positive feelings (Vigour) and a significant decrease in negative feelings (tension anxiety, fatigue, anger, depression, confusion) after forest therapy. There were no significant differences between the groups in the pre-forest-walking values of the subscales of the POMS, with the exception of A-H. Forest walking significantly improved the subscales of the POMS in both groups with no statistical differences between the two groups.	NA
Types of forest interventions: forest therapy program		
Ochiai et al., 2015b [42]; hypertensive middle-aged women (*n* = 6)	SD score: felt more comfortable, relaxed, and natural after forest therapy. POMS score: a significant increase in positive feelings (vigor) and a significant decrease in negative feelings (tension, anxiety, and fatigue) after forest therapy.	
Ochiai et al., 2015a [24]; Pre-hypertensive middle-aged men	SD score: felt more comfortable, relaxed, and natural after forest therapy. POMS score: a significant increase in positive feelings (vigor) and a significant decrease in negative feelings (tension anxiety, confusion, anger, fatigue, and total mood disturbance) after forest therapy.	NA
Song et al., 2017b [43]; pre-hypertensive middle-aged adults; HT: *n* = 9, non-HT: *n* = 17	NA	NA
Sung et al., 2012 [44]; hypertensive elderly people	NA	Quality of life (QoL) scores were obtained at initial visits and at 8-week final visits. MOS SF-36: I, total score increased by 42 compared with the baseline; score increased in PD, MD, HTN by 9, 16, 18, respectively but remained unchanged in SD and GH; C, no change

*LDL* low-density lipoprotein; *HDL* high-density lipoprotein; *RLP* remnant-like particles; *DHEA-S* the serum level of dehydroepiandrosterone sulfate; *hs-CRP* the serum level of high-sensitivity C-reactive protein; *SD* the modified semantic differential; *POMS* the Profile of Mood state; *HF* high-frequency; *LF* low-frequency, *GH* general health; *PD* physical dimensions; *MD* mental dimension; *SD* social dimension; *HTN* hypertension-related dimension

### Quality assessment

The methodological quality of the included studies was rated as moderate to good, with scores ranging from 23-31 out of 42. Most studies with high scores were considered to have clearly stated the aims of the study, and to have followed appropriate data collection strategies and conducted statistical and critical analyses. Most of the studies, however, did not adopt a theoretical framework or a pilot study, and they did not provide information about how the target groups were chosen or how the sample sizes were calculated. The QATSDD scores of each study are shown in the last column of Table 1.

### Study characteristics

Of the included articles, half were conducted in Japan (*n* = 7), while the other half were published in China (*n* = 3), Korea (*n* = 2), Taiwan (*n* = 1), and Finland (*n* = 1). The intervention studies were randomized controlled crossover trials (*n* = 3), randomized controlled trials with parallel groups (*n* = 4), a non-randomized controlled trial (*n* = 1), quasi-experimental studies (*n* = 5), and a quasi-experimental comparative study (*n* = 1).

A total of 951 participants were included in the 14 studies, ranging from 9 to 190 participants in a study. Of these, 281 participants were women and 646 were men. The participants in all of the included studies were adults aged between 19 and 80. Thirteen studies recruited participants with an SBP of 120–180 mmHg or a DBP of 80–110 mmHg, with or without taking anti-hypertensive drugs. One study recruited participants from an information technology company, where 9 were hypertensive and 15 were non-hypertensive, with the latter acting as the comparison group [41]. All of the studies included pre-hypertensive and hypertensive patients.

### Types of forest bathing interventions

The idea of forest bathing has been conceptualized with various types of forest bathing. The types of forest bathing in the included studies consisted of forest walking (*n* = 7); landscape viewing in a forest (*n* = 1); forest walking and viewing (*n* = 2); and a forest therapy program incorporating multiple relaxation activities in a forest environment (*n* = 4).

#### Forest walking

Seven of the included studies adopted forest walking as the intervention. Among them, five studies involved instructed participants not to engage in strenuous physical activities before and during forest walking [23, 33, 34, 36, 42]. The participants were told to keep a steady pace when walking on the forest’s surface where there was no slope or only a slight slope. They were required to refrain from communicating with each other to avoid the effects of conversation, and from drinking alcoholic or caffeinated beverages and smoking. However, in the two studies conducted in China, the participants were walking on paths in the forest that had inclines and declines and were allowed to talk to each other during the walk [43, 44]. The study conducted in Taiwan specified that the focus was on visual, auditory, olfactory, and tactile forms of stimulation during the forest walk [42].

#### Landscape viewing in a forest

Only one study involved the viewing of landscapes in a forest environment as the intervention [35]. The participants were instructed to sit and view the forest landscape for 10 min, without engaging in any physical activity or exercise. During the study period, they were also instructed to avoid smoking and to refrain from drinking alcoholic and caffeinated beverages.

#### Forest walking and viewing

Two studies adopted forest walking and viewing [22, 32]. The study in Finland [22] required the participants to visit three different types of environment (a forest, an urban park, and the city center), with the visits consisting of 15 min of viewing the forest in a sitting position, followed by a 30-min walk. Another study in Japan [32] examined whether a greater expenditure of energy was related to greater changes in blood pressure after forest walking and viewing. The intervention consisted of 90 min of walking in a forest, then lying down to view the forest in a supine position for 20 min. The consumption of alcoholic and caffeinated beverages was also prohibited during the intervention.

#### Forest therapy program

Four studies adopted a forest therapy program comprised of multiple relaxation activities [24, 41, 45, 46]. “Forest therapy” was recognized as being a relaxation and stress management activity carried out in a forest [24, 45]. The forest therapy programs consisted of relaxation activities taking place in the forest environment led by a guide. The participants walked around the assigned area within the assigned period of time, lay on the ground in the forest to rest, and performed deep breathing and meditation exercises [24, 41, 45]. One study also adopted the use of a hammock, blind walking, stretching exercises, and backward walking in the forest program [41]. A study in Korea further included teaching skills on how to manage hypertension, on cultivating the motivation to carry out long-term lifestyle modifications, and relaxation techniques [46]. All of the studies required the participants to avoid smoking tobacco and drinking alcoholic or caffeinated beverages during the forest therapy program.

### Timing of the forest bathing interventions and the weather conditions

All of the forest bathing interventions were conducted during the day on non-working days. Six studies were conducted in the early morning [32, 33, 36, 42–44], two in the afternoon [22, 35], and four for the whole day [24, 41, 45, 46]. One study conducted two sessions of forest bathing in a day—once in the morning and once in the afternoon [34]. One study conducted the intervention either in the morning or in the afternoon [23].

The weather during the forest bathing interventions was either sunny, cloudy, or drizzly, but not rainy. The average temperature ranged from 11 to 25 °C, with humidity ranging from 52.3 to 96%.

### Duration and frequency of the forest bathing interventions

The duration of the forest bathing interventions in the included studies ranged from 10 min to 6 h. In seven studies, the participants walked in a forest environment for 45 to 90 min [22, 23, 32–34, 43, 44]. One study required forest walking for only 17 min [36] and another only required the participants to view forest landscapes for 10 min [35]. Four studies adopted a forest therapy program that lasted for 3 to 6 h [24, 41, 45, 46]. One study in Korea did not report the length of the intervention [46].

The frequency of forest bathing ranged from one to 20 sessions. Nine studies conducted a single session of forest bathing. One crossover RCT study ran two sessions of forest walking in a day [34]. Another RCT study conducted three sessions of forest therapy in three consecutive days [46]. A study in China conducted forest walking for 7 days, one session per day [23]. Another two studies in China provided a total of 20 sessions of forest walks to participants in 20 consecutive days [43, 44].

### Control/comparison intervention

Of the 14 included studies, all but five were intervention studies without a control group for comparison [24, 32, 41, 42, 45].

Four studies adopted walking in an urban area, such as on main streets in a city or in shopping centers, as the control intervention. The participants in the control group walked on flat surfaces or on a slight slope [23, 33, 34, 36]. One study used kinesitherapy as the control intervention, where the participants walked on a highway with inclining and declining slopes around a hospital [44].

In two other studies, one adopted viewing the landscape in an urban area as the control intervention [35], and one adopted walking and viewing the scenery of a city center [22]. One study provided printed educational materials on hypertension management and the self-monitoring of blood pressure as the control intervention [41], while another study reported delivering the usual care as the control intervention [31].

The speed of walking, and the frequency and the duration of the control intervention were the same as those stipulated in the forest bathing intervention in these studies. The weather during the days when the control intervention took place was either sunny or cloudy. The average temperature in the city area was slightly higher than that in the forest environment, ranging from 11 to 30 °C, but the average humidity in the city was similar to that in the forest, ranging from 52.3 to 88% [22, 23, 33–36, 44].

### Time points of the outcome measures

Five of the seven studies on forest walking as the intervention measured physiological data immediately before and after the forest walking activity [23, 33, 42–44]. One other study measured the participants’ blood pressure and pulse rate every 20 min during the intervention, and their state of mood before and after the intervention [34]. The other study measured heart rate and heart rate variability using a wearable electrocardiogram sensing system during the forest walking activity, but assessed the participants’ state of mood only after the intervention [35].

The only study that adopted forest landscape viewing measured the participants’ heart rate and heart rate variability (HRV) during the intervention and their psychological outcomes after the intervention [35]. Among the two studies that adopted forest walking and landscape viewing, one study measured the participants’ blood pressure before the intervention, after their arrival in the forest, after the landscape viewing period, and after their walk in the forest, with the participants wearing an electrocardiogram (ECG) wrist monitors throughout the intervention [22]. The other study measured blood pressure and mood state, and took saliva samples for an amylase analysis before and after the intervention [32].

For the studies that adopted a forest therapy program, the participants’ physiological and psychological outcomes were measured on the day before, during, and after one day of the therapy program [24], or on the day before and 2 h following the forest therapy [45]. The study in Korea measured blood pressure at the initial visit and on day three after the participants completed the program, but salivary cortisol levels and quality of life were measured at the initial visit and at week 8 during the final visit [46]. The other study in Japan measured the participants’ physiological data at 3 days before, during, and 3 and 5 days after the forest therapy program to determine the sustained effect of the program on them [41].

In summary, most studies measured the participants’ physiological and psychological outcomes either on the day before or right before the intervention, and after the completion of the intervention [23, 32, 33, 42–45]. Only two studies measured the participants’ physiological and psychological outcomes in the initial visit through to the post-intervention follow-up to evaluate the sustained effect of forest bathing [41, 46].

### Outcomes of the forest bathing interventions

The outcomes of forest bathing in the fourteen studies can be divided into physiological and psychological measures that are summarized in Table 2 and 3 respectively.

The physiological outcomes included systolic and diastolic blood pressure, pulse rate, heart rate and heart rate variability, salivary or serum cortisol levels, and other outcomes such as cardiac-pulmonary function, cardiovascular and metabolic parameters, and pro-inflammatory cytokine levels (Table 2). The psychological outcomes included mood state, anxiety level, and quality of life (Table 3).

#### Physiological parameters

##### Blood pressure (systolic and diastolic)

Blood pressure was measured as a major outcome of forest bathing induced by the activation of the autonomic nervous system in a state of relaxation [36]. Of the 14 included studies, 11 studies measured blood pressure as the primary outcome.

Six of the studies that adopted forest walking reported significant decreases in systolic blood pressure (SBP) and diastolic blood pressure (DBP) among the participants after the intervention [23, 33, 34, 42–44]. One study reported a remarkable reduction in SBP and DBP of 24.6% and 29.5% respectively among middle-aged Chinese men after undertaking forest walking for 20 sessions on 20 consecutive days [44]. Another study in China also reported that walking in a forest for 20 sessions in 20 consecutive days resulted in a decrease in SBP and DBP of 13.2% and 15.3% respectively among middle-aged men who regularly took antihypertensive drugs [43]. Another study, also conducted in China, reported that walking in a forest for seven sessions on seven consecutive days also produced a significant reduction in SBP and DBP of 5.4% and 7% respectively among hypertensive elderly people compared to those walking in the city [23].

Two forest walking studies, which involved only a single visit to a forest, also reported positive effects [33, 42]. The study in Korea reported a significant reduction in SBP of 8.4% and in DBP of 8.3% in pre-hypertensive elderly people after a single 1-h forest walk compared with a city walk [33]. Another study in Taiwan found that walking in a forest for 2 h resulted in a reduction in SBP of 3.9% and in DBP of 1.5% in pre-hypertensive middle-aged adults and elderly people [42]. However, the study in Japan found no differences between pre-hypertensive elderly women who walked in a forest or in an urban environment for a total of 2 h and 40 min [34].

Among the two studies that adopted forest walking and viewing, one study reported positive effects, with reductions in SBP and DBP of 11% and 5% respectively among middle-aged and elderly people, and a reduction in mean arterial pressure (MAP) of > 5% after 90 min of forest walking and viewing [32]. However, the study in Finland reported no significant changes in SBP and DBP in pre-hypertensive middle-aged women after a 45-min walk in either an urban park or city center [22].

The studies that adopted a forest therapy program with multiple relaxation activities reported a reduction in SBP and DBP after the intervention [41, 45, 46]. One study, after a half-day forest therapy program that lasted for 4 h and 35 min, reported a reduction in SBP and DBP of 11.5% and 9.2%, respectively [45]. Another study reported a 5% decrease in SBP and DBP in middle-aged adults with pre-hypertension after a 6 h forest therapy program [41]. The study that offered a 3-day forest therapy program showed a sustained reduction in SBP of 9% in hypertensive elderly people from the initial measurement to 8 weeks of follow-up assessments [46].

These results indicated that both forest walking and forest therapy programs are effective at reducing the blood pressure of participants, suggesting that they can potentially be used to manage hypertension.

##### Pulse rate

Pulse rate as an index of the activation of the autonomic nervous system was measured in five of the included studies, three of which were on forest walking interventions and two on forest therapy programs.

Studies found that forest walking reduced the pulse rate of pre-hypertensive adults by 3% after 2 h of forest walking [42] and that of hypertensive middle-aged men by 6.9% after two sessions of forest walking [34]. However, a study conducted in China found that a forest walking program consisting of seven sessions held over seven consecutive days did not reduce the pulse rate of elderly people with hypertension [23].

Of the two studies that adopted a forest therapy program and measured pulse rates, one study, conducted in Japan, reported a reduction in pulse rate of 5.4% in hypertensive middle-aged women after a one-day program [24]; but the other study, also conducted in Japan, did not find a significant change in the pulse rate of pre-hypertensive middle-aged adults after 6 h of forest visiting [41].

In summary, forest walking and forest therapy may reduce pulse rates in pre-hypertensive or hypertensive middle-aged adults [24, 34, 42], but not in pre-hypertensive adults or hypertensive elderly people [23, 41].

##### Heart rate and heart rate variability

Heart rate and heart rate variability (HRV) were measured in six of the included studies to quantify autonomic nervous system responses, as indicated by variations in beat-to-beat intervals measured using an electrocardiogram [36].

Of these studies, four examined the effect of forest walking [23, 36, 42, 44]. One study found that the high-frequency component of HRV was 10% higher and heart rates slightly lower in those participants who walked in a forest for 17 min than in those who walked in a city [36]. A study conducted in China reported that the heart rate of middle-aged hypertensive men was 28% lower after 20 sessions in 20 consecutive days of forest walking [44]. However, two other studies found no association between forest walking and heart rate and HRV in middle-aged hypertensive adults and elderly people [23, 42].

With regard to the two remaining studies that assessed heart rate and HRV, the study in Finland reported that forest walking and viewing in an urban forest could lower heart rates by 5.4% and double the high frequency (HF) power of the participants compared to those who walked in the city center [22]. The study in Japan concluded that sitting and viewing in a forest for 10 min resulted in a 30% higher HF power and a 3.5% lower heart rate for the participants than for those in an urban area. There was no significant difference in LF/HF between a forest environment and an urban area [35], whereas the HF was used to estimate parasympathetic nerve activity and the LF/HF ratio was used to estimate sympathetic nerve activity [26, 35].

In summary, forest walking, forest viewing, and forest walking and the viewing of landscapes could increase heart rate variability and reduce the heart rates of pre-hypertensive or hypertensive middle-aged adults [22, 35, 36, 44].

##### Salivary or serum cortisol levels

Salivary cortisol or serum cortisol, a stress hormone, was measured in three studies involving a forest therapy program [24, 45, 46]. Two studies conducted in Japan reported that the forest therapy program was effective at decreasing salivary cortisol levels by 2.5 μg/dL in hypertensive middle-aged women [24] and by 2.63 μg/dL in pre-hypertensive middle-aged men [45] after the intervention. However, the study in Korea did not find any changes in serum cortisol levels after the forest therapy program [41].

These studies suggest that forest therapy programs might have inconsistent effects on reducing sympathetic activity, as measured by the cortisol—stress hormone.

### Cardio-vascular and metabolic parameters, cardiac-pulmonary function, and pro-inflammatory cytokine levels

Of the fourteen included studies, five studies adopted forest walking measured using cardiovascular and metabolic parameters, cardiac-pulmonary function, and pro-inflammatory cytokine levels in participants [23, 33, 34, 43, 44].

Of these, two studies conducted in China found that practicing forest walking in 20 sessions over 20 consecutive days could lead to improvements in high-density lipoprotein (HDL) levels; low-density lipoprotein (LDL) levels; triglycerides (TG); and cardiac function including intima-media thickness (IMT), and brachial-ankle pulse (BaPWV) in hypertensive middle-aged men both in the drug group and the non-drug group [43, 44]. However, one study conducted in Japan reported no change in serum cholesterol, remnant-like particles (RLP), blood glucose, serum insulin, serum dehydroepiandrosterone sulfate (DHEA-S), and high-sensitivity C-reactive protein (hs-CRP) in hypertensive middle-aged men after two sessions of forest walking [34]. By contrast, urinary adrenaline, urinary noradrenaline, and urinary dopamine levels were significantly lower among hypertensive middle-aged men after the two sessions of forest walking, suggesting that forest walking had a relaxing effect on them [34].

Another study in which forest walking was conducted for 7 days [23] showed that levels of endothelin-1 (ET-1), homocysteine (Hcy), renin, angiotensinogen, angiotensin, angiotensin II type 1 receptor (AT1) and angiotensin II (AT2) receptor, and serum interleukin-6 (IL-6) were significantly lower after forest walking. These results indicated that seven sessions over 7 days of forest walking were effective at inhibiting the renin-angiotensin system, reducing sympathetic activity, and inhibiting inflammation in hypertensive elderly people [23].

The cardio-ankle vascular index (CAVI) score, forced expiratory volume in 1 s (FEV1), and forced expiratory volume in 6 s (FEV6), which reflect cardio-pulmonary function, were measured in a study [33]. The result revealed that the CAVI score fell by 0.42+/−0.72, while FEV1 and FEV6 rose by 0.19+/−0.26 and 0.22+/−0.36 respectively after 1 h of forest walking. No significant change in CAVI, FEV1, and FEV6 was demonstrated in 1 h of city walking [33]. The result indicated that forest walking had a positive effective on cardio-pulmonary function.

In summary, it is suggested that forest walking has positive effects on atherosclerotic changes, cardio-pulmonary function, and the inhibition of inflammation.

#### Effect of forest bathing on psychological responses

##### State of mood

Of the included studies, eight studies measured the participants’ state of mood [23, 24, 32, 34–36, 42, 45] using two established instruments: the profile of mood states (POMS) and the semantic differential method (SDM) questionnaire. The former was used to assess the transient, fluctuating, active mood states of the participants and the latter was used to measure their emotions [23, 35, 47, 48]. Four forest walking studies [23, 34, 36, 42], two forest therapy studies [24, 45], and one forest walking and viewing study [32] measured the mood states of the participants using both POMS and SDM. The remaining study that adopted forest viewing measured the mood states of the participants using only the SDM questionnaire [35].

In the eight included studies, the mood state of the participants, as measured using POMS, showed a significant increase in positive mood in terms of feelings of energy and a decrease in negative feelings leading to tension anxiety, fatigue, depression, confusion, and anger among pre-hypertensive or hypertensive middle-aged adults and elderly people after practicing forest therapy, forest walking, and forest walking and viewing [23, 24, 32, 34, 36, 42, 45]. Three studies that measured mood state using SDM reported that pre-hypertensive or hypertensive middle-aged adults felt more comfortable and relaxed after a 1-day forest therapy program [24, 45], and after viewing landscapes in the forest for 10 min [35].

##### Anxiety level

The state-trait anxiety inventory (STAI), which assesses both state and trait anxiety [49], was used in one of the forest walking studies included in this review [42]. The study reported that after 2 h of forest walking, the state anxiety subscale score of pre-hypertensive middle-aged adults and elderly people was a significant 2% lower [42].

##### Quality of life

The Medical Outcomes Study Questionnaire short-form 36 health survey (MOS-SF-36) was used to measure the quality of life (QoL) of the participants after 3 days of a forest therapy program [46]. The MOS SF-36 consisted of five domains measuring general health (GH), the physical dimension (PD), mental dimension (MD), social dimension (SD), and hypertension-related dimension (HTN). The study reported that the total MOS SF-36 score of hypertensive elderly people increased by 42 points compared with the baseline [46]. The result indicated that the PD, MD, and HTN scores increased significantly by 9, 16, 18, but that the GH and SD scores remained at the same level after 3 days of forest therapy [46].

In conclusion, psychological indices including mood state, state anxiety level, and QoL mostly improved after the forest therapy program and forest walking [23, 24, 34–36, 42, 45, 46]. The evidence showed that the forest therapy program had a substantial psychological benefit on middle-aged adults and elderly people with pre-hypertension or hypertension [46].

## Discussion

The focus in this literature review was on exploring the physiological and psychological benefits of forest bathing among middle-aged adults and elderly people suffering from pre-hypertension or hypertension. Another aim was to identify the types of forest bathing interventions that were effective, the duration and frequency of the interventions that would be required to produce positive results, the outcomes of measures providing evidence of the effectiveness of an intervention, and the appropriate measuring time points, to provide guidelines or directions for future research on forest bathing interventions.

This review identified forest walking, forest landscape viewing, forest walking and viewing, and a forest therapy program with multiple relaxation activities, as the four main types of forest bathing interventions. Of the four, forest walking and forest therapy programs demonstrated the most significant physiological and psychological effects on pre-hypertensive or hypertensive middle-aged adults and elderly people [23, 24, 33, 34, 36, 41–46].

The results showed that practicing forest walking and participating in a forest therapy program could reduce the blood pressure [23, 33, 41–46], lower the pulse rate [24, 34, 42], and increase the power of the HRV of participants [36, 44]. In addition, forest walking could improve cardiovascular and metabolic function, cardiac-pulmonary function, and inhibit inflammation in middle-aged and elderly individuals with pre-hypertension or hypertension [23, 33, 34, 43, 44]. The forest therapy program had positive effects on stress relief, as evidenced by the lowering of salivary and serum cortisol levels among the participants [24, 45, 46].

Forest walking and forest therapy were also demonstrated to have a psychologically relaxing effect on hypertensive individuals [23, 24, 32, 34–36, 42, 45]. Forest walking and forest therapy could reduce the negative emotions of anxiety and depression among the participants [23, 24, 34, 36, 42, 45]. Practicing forest therapy could also improve their quality of life [46], and practicing forest walking for 2 h could reduce their anxiety levels [42].

Although practicing 20 sessions in 20 consecutive days of forest walking led to significant improvements in cardiac function and to a remarkable reduction in blood pressure among hypertensive middle-aged men, its effectiveness on psychological relaxation was uncertain [43, 44]. Nevertheless, studies involving a single 2-h forest walk or a 4-h forest therapy program also reported short-term effects on a reduction in blood pressure, a lowering of pulse rate, a boost in positive mood, and a reduction in anxiety in pre-hypertensive or hypertensive middle-aged adults and elderly people [24, 42, 45].

Few studies examined the sustained effects of forest bathing on both the emotional and physical condition of individuals [41, 46]. One study reported that the effect of a reduction in blood pressure lasted for 5 days after a 1-day forest therapy program [41]. Another study found that a decrease in blood pressure and an improvement in quality of life could be sustained for 8 weeks after a 3-day forest therapy program [46]. Most of the included studies measured physiological and psychological outcomes either on the day before or immediately before, and after the completion of the interventions [23, 32, 33, 42–45]. Only two studies measured the psychological and physiological outcomes of forest bathing among pre-hypertensive or hypertensive adults from the initial visit to the post-intervention follow-up [41, 46].

### Implications for practice

Exposure to prolonged stress is one of the major causes of high blood pressure. In recent decades, forest bathing has been proposed as a health promotion strategy for preventive medicine to lower blood pressure and reduce stress, and to induce a positive mood [23, 24, 32–34, 41, 43, 45, 46]. Forest bathing, in which people connect with nature by opening all the senses that they possess, could induce relaxation and strengthen the immune system [16, 20, 21].

As the natural environment is regarded as one of the most vital of health resources [50], the Japanese government has incorporated it into the country’s health program [20]. In America, relaxing in nature has been recommended as a way of managing stress to prevent hypertension and heart disease [38]. These findings have demonstrated that forest bathing could be an enjoyable complementary approach to controlling hypertension. Healthcare professionals should consider the practice of forest bathing as a “preventive medicine” to reduce stress and lower blood pressure in those suffering from hypertension.

### Recommendations for future research

Evidence-based guidelines on forest bathing as an intervention should be developed. High-quality studies should be conducted on forest walking or forest therapy programs as a health promotion approach to evaluate the health benefits for pre-hypertensive and hypertensive clients, to establish strong evidence of their clinical effects.

For further studies, it is recommended that researchers examine both the emotional and physiological effects of forest therapy or forest walking on pre-hypertensive or hypertensive middle-aged individuals. Both physiological and psychological impacts, such as on SBP, DBP, pulse rate, heart rate variability, salivary cortisol concentration, mood states, and anxiety level, which reflect the stress-relieving effects of forest therapy or forest walking, should be measured to examine the potential health benefits of such programs on hypertension [51].

There have been few studies on the sustained emotional and physical effects of forest bathing on individuals [41, 46]. Therefore, the existing evidence on the therapeutic effects of forest bathing is insufficient to establish clinical guidelines on its use as a health promotion strategy or disease prevention activity.

## Limitations

Although it was observed in this review that forest bathing had positive therapeutic effects on pre-hypertensive and hypertensive adults, there are several limitations to this review. First, only studies on forest bathing interventions published in English and Chinese were examined; those in other languages were not identified. As forest bathing has been accepted in Japan as a health strategy for the prevention of illness [20], a number of papers on forest bathing have been published in Japanese, leading to a possible language bias. Second, conference proceedings, abstracts, and dissertations were excluded from this review. Reviewing only published articles may be a potential source of bias, since studies with statistically significant results are more likely to be accepted for publication.

## Conclusions

Practicing forest bathing was found to lower blood pressure and heart rates, induce a positive mood, and reduce anxiety levels. The evidence presented in the studies that were reviewed here indicates that forest walking and forest therapy programs are the most effective types of forest interventions.

Two hours of forest walking during a single visit or 4 h of a forest therapy program had physiologically and psychologically relaxing effects on middle-aged adults with elevated blood pressure. Measurements should be obtained at baseline, before and after the intervention, and at the 8-week post-intervention follow-up. It is suggested here that an intervention study be conducted to examine the possible benefits of forest bathing among middle-aged adults in Hong Kong with pre-hypertension or hypertension.

## Data Availability

Data sharing is not applicable to this article as no datasets were generated or analyzed during the current study.

## Abbreviations

CAJ: China Academic Journals
CNKI: Full-text database
HRV: Heart rate variability
MOS SF-36: The Medical Outcomes Study Questionnaire short-form 36 Health survey
HF: High frequency
LF: Low-frequency
HRV: Heart rate variability
QATSDD: Quality assessment tool for studies with diverse designs
FMD: Flow-mediated dilation
NMD: Nitro-glycerine mediated dilation
IMT: Carotid intima-media thickness
BaPWV: Brachial-ankle pulse wave velocity
TG: Triglycerides
LDL: Low-density lipoprotein
HDL: High-density lipoprotein
RLP: Remnant-like particles
DHEA-S: The serum level of dehydroepiandrosterone sulfate
hs-CRP: The serum level of high-sensitivity C-reactive protein
GH: General health
PD: Physical dimension
MD: Mental dimension
SD: Social dimension
HTN: Hypertension-related dimension
CAVI: Cardio-ankle vascular index
FEV1: Forced expiratory volume in 1 s
FEV6: Forced expiratory volume in 6 s
ET-1: Endothelin-1
Hcy: Homocysteine
RAS: Renin-angiotensin system
SD: The modified semantic differential
POMS: The profile of mood state
ECG: Electrocardiogram
STAL: The state-trait anxiety inventory
RCT: Randomized control trials
SBP: Systolic blood pressure
DBP: Diastolic blood pressure
MAP: Mean arterial pressure
